# 中国驱动基因阳性非小细胞肺癌脑转移临床诊疗指南（2025版）

**DOI:** 10.3779/j.issn.1009-3419.2024.102.42

**Published:** 2025-01-20

**Authors:** 中国医药教育协会肺癌医学教育专业委员会, 北京医学奖励基金会肺癌医学青年专家委员会脑转移协作组

**Keywords:** 肺肿瘤, 脑转移, 驱动基因, 靶向治疗, 临床诊疗指南, Lung neoplasms, Brain metastasis, Actionable gene alterations, Targeted therapy, Clinical practice guidelines

## Abstract

脑转移已成为非小细胞肺癌（non-small cell lung cancer, NSCLC）患者治疗全程管理中的重大挑战，在携带驱动基因突变的患者中尤为突出。传统治疗如放射治疗和外科手术的临床获益有限，且常伴随认知功能障碍和生活质量下降。近年来，针对表皮生长因子受体（epidermal growth factor receptor, *EGFR*）、间变性淋巴瘤激酶（anaplastic lymphoma kinase, *ALK*）等靶点的新型小分子酪氨酸激酶抑制剂不断涌现，有效穿透血脑屏障的同时提升了颅内药物浓度、改善患者预后，从而打破了NSCLC脑转移既往的治疗格局。因此，中国医药教育协会肺癌医学教育专业委员会、北京医学奖励基金会肺癌医学青年专家委员会脑转移协作组联合发起并制定了《中国驱动基因阳性非小细胞肺癌脑转移临床诊疗指南（2025版）》。本指南通过整合最新研究成果与临床经验，基于多学科诊疗原则，涵盖驱动基因阳性NSCLC脑转移的诊断、治疗时机及系统和局部治疗选择等内容。同时，指南提出了针对不同驱动基因类型的个体化治疗策略，旨在为临床医师提供参考，提升中国NSCLC脑转移的整体诊疗水平。

## Abstract (en)

Brain metastasis has emerged as a significant challenge in the comprehensive management of patients with non-small cell lung cancer (NSCLC), particularly in those harboring driver gene mutations. Traditional treatments such as radiotherapy and surgery offer limited clinical benefits and are often accompanied by cognitive dysfunction and a decline in quality of life. In recent years, novel small molecule tyrosine kinase inhibitors targeting epidermal growth factor receptor (*EGFR*), anaplastic lymphoma kinase (*ALK*), and other pathways have been developed, effectively penetrating the blood-brain barrier while enhancing intracranial drug concentrations and improving patient outcomes. This advancement has transformed the treatment landscape for brain metastases in NSCLC. Consequently, the Lung Cancer Medical Education Committee of the Chinese Medical Education Association and the Brain Metastasis Collaboration Group of the Lung Cancer Youth Expert Committee of the Beijing Medical Reward Foundation have jointly initiated and formulated the *Clinical Practice Guidelines for the Management of Brain Metastases from Non-small Cell Lung Cancer with Actionable Gene Alterations in China (2025 Edition)*. This guideline integrates the latest research findings with clinical experience, adhering to multidisciplinary treatment principles, and encompasses aspects such as diagnosis, timing of intervention, and systemic and local treatment options for driver gene positive NSCLC brain metastases. Additionally, it proposes individualized treatment strategies tailored to different driver gene types, aiming to provide clinicians with a reference to enhance the overall diagnostic and therapeutic standards for NSCLC brain metastases in China.

脑转移（brain metastases）已成为非小细胞肺癌（non-small cell lung cancer, NSCLC）诊疗实践中日益严峻的挑战^[1]^。尽管存在血脑屏障（blood-brain barrier, BBB），脑仍然是NSCLC常见的转移部位之一。流行病学数据^[2,3]^显示，约10%的NSCLC患者在初诊时即存在脑转移，而26%-53%的患者在病程中进展为脑转移。大样本多瘤种长期队列随访发现，肺癌的脑转移远高于其他常见恶性肿瘤^[2]^。携带表皮生长因子受体（epidermal growth factor receptor, *EGFR*）突变、间变性淋巴瘤激酶（anaplastic lymphoma kinase, *ALK*）重排等驱动基因阳性的NSCLC患者，相比驱动基因阴性患者，表现出更高的脑转移风险和更早的发病时间^[4]^。有研究报道，*EGFR*突变型NSCLC患者的3年累积脑转移率可达29.4%-60.3%^[5]^；*ALK*阳性患者从确诊到脑转移的中位时间仅为88 d^[6]^。中国NSCLC患者5年生存率在过去十年间从13.8%上升至23.7%^[7]^，考虑到治疗后的驱动基因阳性患者生存期普遍更长^[8]^，因此我国未来NSCLC脑转移的负担可能将进一步加重。

NSCLC发生脑转移的机制尚未完全明确，可能是起始自上皮间充质转化（epithelial-mesenchymal transition, EMT），导致侵袭性增强，经血液循环穿过BBB后，完成颅内种植^[9]^。脑转移发生后，NSCLC患者的总生存期显著降低，经治后中位生存期仅为7-12个月^[10,11]^，传统上，由于BBB的存在限制了化疗药物的颅内渗透，NSCLC脑转移的治疗主要依赖于放射治疗和外科手术等局部治疗方法。然而，越来越多的证据表明全脑放射治疗（whole-brain radiotherapy, WBRT）的临床获益有限，单纯立体定向放射外科（stereotactic radiosurgery, SRS）或WBRT治疗脑转移患者的中位生存期仅为4.6个月^[12]^，且常伴随认知功能障碍和生活质量下降^[13]^。

近年来，针对*EGFR*、*ALK*、c-ros肉瘤致癌基因1（ROS proto-oncogene 1, *ROS1*）等靶点的新型小分子酪氨酸激酶抑制剂（tyrosine kinase inhibitors, TKIs）的出现为脑转移患者带来了新的希望。这些新药凭借其分子量小和脂溶性强等特征，能够有效穿透BBB，甚至不受BBB外排作用的影响^[14]^，从而大幅提升了颅内药物治疗浓度^[15]^；进而显著改善了颅内客观缓解率（objective response rate, ORR）并延长了无进展生存期（progression-free survival, PFS）^[16⇓-18]^，部分患者甚至在靶向单药治疗的情况下达到颅内完全缓解（complete response, CR）^[19]^；加之EGFR-TKIs药物的“剂量倍增”疗法和双靶药物[如EGFR/间质-上皮细胞转化因子（mesenchymal-epithelial transition factor, MET）、程序性死亡受体1（programmed cell death 1, PD-1）/血管内皮生长因子（vascular endothelial growth factor, VEGF）双抗]的出现，*EGFR*敏感突变特别是靶向耐药的NSCLC脑转移患者的预后又有进一步的提升^[20,21]^。由此可见，对于驱动基因阳性的NSCLC脑转移患者，有必要重新评估颅脑局部治疗的使用时机以及初始治疗中靶向药物的选择。

鉴于此，中国医药教育协会肺癌医学教育专业委员会、北京医学奖励基金会肺癌医学青年专家委员会脑转移协作组（以下简称“协作组”）特别组织专家制定了《中国驱动基因阳性非小细胞肺癌脑转移临床诊疗指南（2025版）》。本指南旨在提供基于多学科诊疗（multi-disciplinary treatment, MDT）原则的规范化方案，专门针对驱动基因阳性NSCLC脑转移患者（特别是靶向治疗），通过规范NSCLC脑转移的诊疗实践，改善患者预后，同时为广大肿瘤专科医师提供参考，从而提升中国在NSCLC诊疗方面的整体水平。

## 1 方法和讨论范围

本指南的撰写主要基于截至2024年10月前发表的临床循证，兼顾协作组专家的意见和经验。协作组成员在充分总结驱动基因阳性NSCLC脑转移循证基础上，提炼出若干推荐意见，强调其实用性和前沿性，并兼顾可操作性与可及性。

本指南中的每条推荐都给出了临床经验和循证两个方面的评价，这些评价在GRADE（Grading of Recommendations, Assessment, Development and Evaluation）标准^[22]^基础上做了简化（表1），以适应亚组分析为主的NSCLC脑转移循证特点。临床经验的分级由协作组成员投票达成共识；不同药物之间的优选，协作组根据直接证据和研究类型，以及参考药物的BBB穿透力、研究是否有总生存期（overall survival, OS）或CR结果进行综合判断。

**表1:** 推荐强度/证据等级标准

推荐强度	基于临床经验的分级	标准	证据等级	标准
1	强推荐	临床实践中非常有必要， 根据经验临床获益≥风险	A	基于至少一项III期RCT^a^
2	中等推荐	临床实践中有必要， 根据经验临床获益≥风险	B	基于至少一项III期RCT亚组分析^b^， 或多项I、II期前瞻性研究及其亚组分析^c^
3	弱推荐	临床实践中可能有必要， 缺乏充分的临床经验	C	基于一项I、II期亚组分析，或观察性、回顾性研究、 病例报道
			D	基于专家意见，暂无证据

^a^：有对照组，或放化疗或同类药物或安慰剂；^b^：预设/非预设亚组；^c^：包括开放标签、单臂。 RCT：随机对照研究。

本指南重点聚焦驱动基因阳性NSCLC脑转移，其中脑转移是指NSCLC肿瘤组织在脑实质（包括大脑、小脑和脑干）内的转移，暂不包括脑膜，因为脑膜的循证依据较少，现有的国内外神经系统转移瘤指南已经推荐的诊疗格局并未发生改变。NSCLC驱动基因目前可干预的且有脑转移相关循证的共9个，针对这些靶点脑转移灶的靶向治疗是本指南的重点讨论内容。

## 2 驱动基因阳性NSCLC脑转移的诊断

驱动基因阳性的NSCLC脑转移的诊断过程主要包括两个关键部分：明确颅脑转移瘤的存在与来源、驱动基因的分型。前者主要依赖磁共振成像（magnetic resonance imaging, MRI）等影像学检查，而后者主要借助于组织和体液的分子检测。

### 2.1 症状体征

NSCLC在脑转移的早期阶段往往没有明显的症状和体征，其症状和体征取决于脑转移灶的数量和位置，大多为非特异性。常见症状为头痛伴随恶心和喷射性呕吐，是由于肿瘤生长及其周围水肿导致的颅内压增高或直接影响脑脊液（cerebrospinal fluid, CSF）循环引起的，头痛往往呈双侧弥漫性头痛，疼痛性质往往为钝痛和跳痛^[23]^。颅内肿瘤占位病变也可以引起轻偏瘫、语言障碍和视野缺损，通常是亚急性和逐渐加重的。此外，15%-25%的脑转移患者会出现癫痫^[24]^。

### 2.2 影像诊断

#### 2.2.1 MRI与计算机断层扫描（computed tomography, CT）

NSCLC脑转移的颅脑MRI常见典型表现为T1WI低信号、T2WI高信号、弥散加权成像（diffusion weighted imaging, DWI）高信号、明确强化、伴瘤周水肿；少见表现为T1WI、T2WI等/低信号、DWI等/低信号、低强化等非典型表现^[25]^。通过提高MRI场强、延长注入造影剂后成像时间或增加造影剂量可提高脑转移灶的检出率^[26,27]^。颅脑增强MRI（层厚≤1.5 mm的增强T1WI和增强T2-FLAIR序列）可以增加诊断的敏感性和特异性^[28]^，必要时颅脑增强MRI联合功能成像（DWI、脑灌注成像、脑波谱成像）有助于诊断、鉴别诊断以及疗效评价^[29,30]^。有研究^[31,32]^认为不同驱动基因突变类型NSCLC脑转移的MRI影像表现可能存在一定差异。

NSCLC脑转移的颅脑CT，常见表现为低密度或等密度影，增强CT病灶强化明显。与颅脑增强MRI相比，其对脑转移灶检出的敏感性较低，特别是对早期、小的转移病灶。不过，颅脑CT有MRI不具备的优势：扫描速度快，对瘤内出血及脑疝等颅内压增高急症的识别更为准确和迅速。

因此，颅脑增强MRI检查应作为NSCLC脑转移的首选诊断方法。对有MRI禁忌证的患者，或出现脑转移急症时，可考虑CT替代。

值得注意的是，7%的III期NSCLC患者被MRI诊断脑转移阴性后的1年内进展至阳性^[33]^。因此颅脑增强MRI检查应该是一个动态过程，特别是对易发生脑转移的驱动基因阳性NSCLC患者。

**推荐1：颅脑增强MRI检查是判断脑实质转移的首选影像检查（1A）。**

**推荐2：当患者无法行颅脑增强MRI或出现脑转移急症时，颅脑增强CT可作为替代影像检查手段（1D）。**

#### 2.2.2 PET-CT/MRI

正电子发射计算机断层扫描（positron emission tomography, PET）与CT/MRI的结合，既将解剖与功能相结合，又从分子层面反映脑组织的生理、病理及代谢信息。PET-CT检测脑转移敏感性不如MRI^[34]^，容易漏诊，故不建议使用PET-CT/MRI作为NSCLC的脑转移初始诊断方式；即使已完成全身PET-CT/MRI检查，如不能确定脑转移，仍建议行颅脑薄层增强MRI，以确保未漏诊任何潜在的脑转移灶^[35]^。

但值得注意的是，PET对明确手术指征、治疗后疗效评价及确定脑转移原发灶有一定的临床价值。例如，PET-CT/MRI可鉴别术后或放疗后肿瘤残存与炎性反应；也有助于区分由放疗诱发的脑损伤与脑转移瘤的“真性进展”，灵敏度为95%，特异度高达100%^[36⇓⇓-39]^。

**推荐3：对于脑转移高危驱动基因阳性NSCLC患者，如果全身PET-CT/MRI检查未发现脑转移，仍建议行颅脑薄层增强MRI排除（1D）。**

### 2.3 病理与分子分型

肺癌脑转移组织通常呈现出原发灶的组织学形态，通常局限于脑实质的肿瘤包膜内，个别病例可能表现出浸润性特征^[40]^。目前，如果原发性肺癌明确，影像学可以明确脑转移，病理检查则并不是必要确诊步骤，但可以作为诊断和评价治疗的辅助手段，特别是在原发灶和颅内转移基因突变表型不一致的情况下。系统性回顾研究^[41]^显示，约10%的患者在肺部原发灶中存在*EGFR*或大鼠肉瘤病毒癌基因同源物突变（Kirsten rat sarcoma viral oncogene homolog, *KRAS*），而在颅内转移灶中这些突变可能消失（表现为野生型）。如果治疗过程中原发灶与颅内转移灶疗效不一致，这时可考虑进一步明确颅内转移灶的基因突变表型。

针对NSCLC患者不同驱动基因的分子检测方法，目前尚无普适的标准检测方案。美国国立综合癌症网络（National Comprehensive Cancer Network, NCCN）指南指出^[42]^，所有晚期或转移性NSCLC患者应接受*EGFR*、*ALK*、*MET*ex14跳跃突变、转染重排基因（rearranged during transfection, *RET*）、*ROS1*、*KRAS*、V-Raf鼠肉瘤病毒癌基因同源体（V-Raf murine sarcoma viral oncogene homolog B, *BBRAF*）、神经生长因子受体酪氨酸激酶（neurotrophic tyrosine receptor kinase, *NTRK*）（包括*NTRK 1*、*2*、*3*）和人类表皮生长因子受体2（human epidermal growth factor receptor 2, *HER2*）（*ERBB2*）共9个靶点的分子检测；指南还推荐通过组织活检和/或血浆检测进行分子检测，无论是同时还是顺序进行的组织和血浆检测组合均可接受。如果一种方法结果为阴性，可以使用补充方法进行交叉验证。具体检测可参考《原发性肺癌罕见靶点靶向治疗中国临床诊疗指南（2024版）》^[43]^、《CSCO非小细胞肺癌》^[44]^等指南的相关章节。

近年来，脑脊液的液体活检已成为NSCLC脑转移诊断的重要手段之一。小样本研究^[45]^提示，脑脊液循环肿瘤DNA（circulating tumor DNA, ctDNA）评价驱动基因更能代表脑转移灶的基因突变情况，而且CSF中的驱动基因突变检出率高于血浆，可作为血浆分子检测的有效补充，也能为靶向药耐药后提供治疗依据。

**推荐4：影像学考虑脑转移，且肺部原发肿瘤明确时，脑组织病理检查不是肺癌脑转移的必要确诊步骤（1D）。**

**推荐5：NSCLC脑转移患者应进行驱动基因的分子检测（1A），基于组织、血浆或脑脊液标本检测的结果均可指导临床治疗（1D）。**

## 3 驱动基因阳性NSCLC脑转移的治疗

NSCLC脑转移的治疗可以大致分为神经症状控制和针对脑转移瘤处理两部分。前者包括降颅压、改善水肿、癫痫防治、改善神经症状等；后者指局部手术、放疗和全身用药（靶向治疗、化疗、免疫治疗等）。本指南主要讨论针对脑转移瘤的处理。

欧洲神经肿瘤学协会（European Association of Neurooncology, EANO）-欧洲医学肿瘤学会（European Society of Medical Oncology, ESMO）实体瘤脑转移指南（2021版）^[25]^认为，脑转移治疗的目标是预防或延缓神经功能恶化，并在可接受的生活质量下延长生存期。对于驱动基因阳性的NSCLC脑转移患者，上述目标也同样适用，特别是当病灶较小且为寡转移的患者，可能会有长生存甚至治愈的机会，因此应在防治神经并发症的同时，积极进行颅内转移瘤和原发肺部肿瘤的综合治疗。

### 3.1 一般原则

#### 3.1.1 局部干预与全身用药时机和顺序的选择

既往脑转移的治疗以局部治疗为主，但对于携带驱动基因（如*EGFR*、*ALK*等）的患者，由于新一代靶向治疗相比传统化疗或较早上市的靶向药物，无论是单药还是联合放化疗，均可使NSCLC脑转移的颅内控制和PFS得到显著提升^[46⇓-48]^。鉴于此，协作组重新评估了颅脑局部干预与全身用药时机和顺序。

在无脑转移相关症状的情况下，协作组认为在充分MDT讨论后可推迟针对颅内转移灶的放疗或手术干预，优先使用靶向治疗（特别是*EGFR*、*ALK*等循证充分的靶点，详见“3.2 靶向治疗”章节），直至颅内病情进展，理由如下：

首先，包括NSCLC在内的大多数脑转移患者，直接死因并不是脑转移病灶^[25]^。因此，相对于肺癌原发灶，无需急症干预的脑转移病变治疗相对并不急迫，特别是在无神经症状的患者中，治疗的重点应放在原发肿瘤的系统治疗上。

其次，以第三代EGFR-TKIs为代表的新型靶向药物已经被证实其颅内活性显著提升，克服了化疗药物难以穿透BBB的不足，加之有临床证据^[46⇓-48]^表明，单药或作为二线联合可使*EGFR*、*ALK*、*RET*等阳性的颅内病变达到CR，还能同步治疗原发肺部癌变组织，进而使初始颅内局部治疗的必要性降低，因此有相应靶点的患者优先采用靶向治疗更为合理。

再次，尽管有研究^[49⇓⇓-52]^支持NSCLC脑转移患者预先放疗（up-front radiotherapy）后贯序使用TKIs（通常为放疗后4周）的策略，但应该注意到，这些研究多以回顾性研究为主，而非随机对照研究。进入靶向治疗时代后，证据显示EGFR-TKIs单药治疗相比放疗±化疗，颅内PFS显著延长，尤其是存在多个脑转移灶的患者^[53]^；此外，EGFR-TKIs同步联合WBRT或在TKIs治疗前使用放疗生存获益并没有进一步增加^[54,55]^，因此美国临床肿瘤协会（American Society of Clinical Oncology, ASCO）、神经肿瘤学会（American Society of Neurooncology, SNO）和美国放射治疗及肿瘤学会（American Society for Radiation Oncology, ASTRO）脑转移治疗指南（2021）支持携带癌症驱动基因突变的NSCLC无症状脑转移患者可以延迟局部治疗，先给予靶向治疗^[56]^。ASTRO脑转移放疗指南（2022版）^[57]^也认为，对于符合全身治疗条件的无症状脑转移患者，安全推迟局部治疗是可考虑的。此外，WBRT或SRT还存在病灶数量和大小相关的适应证限制；而且还存在无法忽视的认知障碍不良反应^[58]^，同步靶向治疗和放疗的神经毒性可能更为严重^[59]^。

尽管越来越多的证据支持将靶向治疗作为一线初始方案，但协作组仍建议在制定方案前，应进行充分的MDT讨论^[60]^，遵循个体化的原则，兼顾疗效、生活质量和患者意愿。

**推荐6：在制定驱动基因阳性NSCLC脑转移治疗方案前，推荐进行MDT讨论，遵循个体化的原则，兼顾疗效、生活质量和患者意愿（1D）。**

**推荐7：对于无症状的脑转移患者，可考虑推迟针对颅内转移灶的放疗或手术干预，优先使用靶向治疗（1A）。**

#### 3.1.2 靶向药物耐药颅内进展的处理

协作组在本指南制定过程中，汇总了36项靶向治疗NSCLC研究，在不考虑联合或单药、一线二线用药的情况下，经治后的中位颅内PFS均值约为12.93个月（95%CI: 10.94-14.92），提示一半的NSCLC脑转移患者在服用靶向药物约1年后会发生颅内进展。协作组建议在服用靶向药物1年后应积极监测颅内进展情况，对靶向治疗相对不敏感的*EGFR*非经典突变、*HER2*、*BRAF* V600等靶点，评估时间应更为提前（具体见“3.2 靶向治疗”章节）。

初始靶向药物治疗后，如颅内病变进展，首先要明确是否伴随颅外进展（图1），对于颅外病变控制良好而颅内进展患者，可以继续服用原靶向药物，针对脑转移灶行局部处理（优先推荐放疗）。部分患者可能是由于颅内靶向药物浓度较低，有部分第三代EGFR-TKIs“剂量倍增”后，颅内病变仍能得到一定程度的改善^[20]^；但“剂量倍增”疗法循证仅局限于部分药物，是否具有普适性需要更多研究证实（详见“3.2.1 *EGFR*突变”章节）。如果伴随颅外进展，需要结合颅内进展是否伴随相关症状决定局部治疗是否介入，推荐再次进行基因检测，根据检测结果指导全身治疗。

**图1:**
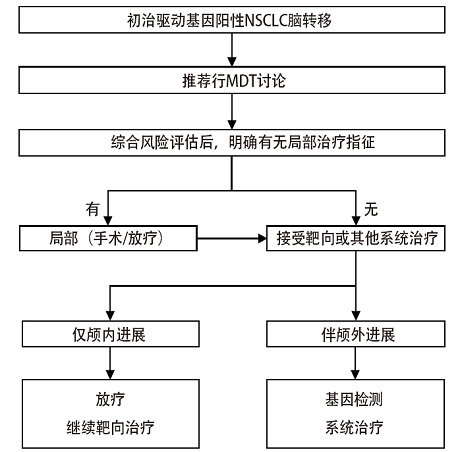
驱动基因阳性NSCLC脑转移一般治疗流程 NSCLC：非小细胞肺癌；MDT：多学科诊疗。

应当注意的是，各个靶点处理的方式不尽相同，上述提出的各种治疗方案（图1）仅作为一般流程参考，具体详见各靶点治疗章节，化疗具体方案可参考相关肺癌指南。

### 3.2 靶向治疗

NSCLC中可干预的靶点通常按发生率分为常见（≥5%）和罕见（<5%）两类。

*EGFR*突变是NSCLC中最常见的驱动基因突变，也是循证最夯实的NSCLC靶点，靶向治疗ORR高，特别是在第三代TKIs出现后疗效提升明显，各种耐药、继发突变的处理方法也逐渐成熟。*ALK*、*ROS1*、*NTRK*和*RET*属于基因重排或融合，单一靶向治疗预后都相对良好，生存期较长，尽管出现耐药，也多为该靶点依赖性继发突变；同时一线、二线用药的证据丰富，是相应驱动基因阳性脑转移治疗的基石，也是本指南讨论和推荐的重点。

对于*KRAS* G12C、*BRAF* V600、*EGFR*和*HER2 *ex20ins或*MET*ex14跳跃突变，相应靶向药物的ORR偏低，而且该类NSCLC脑转移人群的循证样本量偏低，特别是一线靶向治疗证据不足。例如*HER2*和*KRAS* G12C突变的靶向治疗循证主要来自二线用药；*MET*ex14跳跃突变的靶向治疗虽有用于一线，但疗效不尽如人意；*BRAF* V600突变多采用联合用药方案。因此，协作组认为该类靶点与其他NSCLC相关指南的推荐区别不大，本指南只做重点循证更新。

#### 3.2.1 *EGFR*突变

*EGFR*突变根据受体结构不同可以分为四种亚型：经典型、T790M型、ex20ins突变、PACC型突变^[61]^。经典型突变包括常见的21外显子L858R点突变和19外显子缺失（exon 19 del）突变。此类突变对于各类EGFR-TKIs均有较好疗效。另外三类亚型一线EGFR-TKIs治疗数据有限。因此本指南仅针对*EGFR*经典突变和*EGFR* ex20ins突变脑转移患者治疗进行推荐。

##### 3.2.1.1 *EGFR*经典型突变的靶向治疗

EGFR-TKIs问世已有20年，历经三代药物发展，临床循证非常夯实。以奥希替尼（Osimertinib）为代表的第三代EGFR-TKIs的疗效已全面超越第一代。目前将大部分第三代EGFR-TKIs和第一代药物做过临床III期一线用药的疗效比较^[62,63]^，结果一致表明第三代药物PFS较第一代有显著改善。此外，基于APPLE等研究^[64]^结果，第一代EGFR-TKIs出现T790M继发突变后改换第三代EGFR-TKIs的贯序疗法，相比直接使用第三代EGFR-TKIs，OS相似，但贯序治疗组颅内PFS更高，因此对经典型突变患者形成了一线优选第三代EGFR-TKIs的治疗格局，但第三代EGFR-TKIs之间缺少头对头的比较研究。

针对脑转移患者，靶向*EGFR*不仅能够控制颅内病变，而且在一定条件下优于局部治疗。BRAIN研究^[53]^是第一个在*EGFR*突变阳性NSCLC使用靶向治疗（埃克替尼，Icotinib）与放疗（全脑放射）+化疗两种方案相对比治疗NSCLC脑转移的前瞻性研究，结果发现靶向治疗组中位颅内PFS为10个月，显著优于放化疗组（4.8个月，*P*=0.014），尽管12个月时两组生存率相似（79% *vs* 76%）。此外，BRAIN研究中，靶向治疗3-4级不良反应发生率显著低于全脑放射组（8% *vs* 38%），可见安全性更好。EVEREST研究是第一个针对*EGFR*阳性、未接受过颅内放疗的NSCLC脑转移人群进行的大样本、随机对照、III期研究^[65]^，结果显示佐利替尼（Zorifertinib）一线治疗相较于第一代EGFR-TKIs[吉非替尼（Gefitinib）或厄洛替尼（Erlotinib）]，显著延长颅内PFS（15.2 *vs* 8.3个月，HR=0.517，*P*<0.0001），基于研究者使用RANO-BM评估的颅内PFS（17.9 *vs *11.1个月，HR=0.627，*P*=0.0018）、颅内ORR和DOR均优于第一代EGFR-TKIs，3年总体生存率为43.4%；对L858R突变或颅内病灶数>3个亚组也有同样的获益。

其他主流第三代EGFR-TKIs的临床III期研究均有脑转移亚组循证支持，相比第一代EGFR-TKIs，第三代EGFR-TKIs具有较高的颅内ORR和延长PFS的作用（详见表2^[53,65⇓⇓⇓⇓-70]^）。

**表2:** 不同EGFR-TKIs在EGFR阳性NSCLC脑转移人群中的循证

研究项目	研究类型	治疗药物	脑转移样本量^a^	CNS ORR	CNS CR	颅内PFS（月，HR，95%CI）
FLAURA2^[66]^	III期 RCT	奥希替尼+铂类-培美曲塞 vs 奥希替尼	78^b^	88% vs 87%	48% vs 16%	30.2 vs 27.6 HR=0.58 (0.33-1.01)
FLAURA^[67]^	III期 RCT	奥希替尼 vs 吉非替尼/厄洛替尼	41^b^	91% vs 68%	23% vs 0%	NR vs 13.9 HR=0.48 (0.26-0.86); P=0.014
AENEAS^[68]^	III期 RCT	阿美替尼 vs 吉非替尼	61^b^	82.8% vs 75%	-	29.0 vs 8.3 HR=0.319 (0.176-0.580); P<0.0001
FURLONG^[69]^	III期 RCT	伏美替尼 vs 吉非替尼	133^b,c^	91% vs 65%	9% vs 0%	20.8 vs 9.8 HR=0.40 (0.23-0.71); P=0.0011
IBIO-103^[70]^	III期 RCT	贝福替尼 vs 埃克替尼	92^b^	92.3% vs 55.6%	7.7% vs 5.6%	24.9 vs 15.2 HR=0.85 (0.41-1.76); P=0.6606
EVEREST^[65]^	III期 RCT	佐利替尼 vs 吉非替尼或厄洛替尼	439	75.6% vs 62.3%	-	17.9 vs 11.1^d^ HR=0.467 (0.352-0.619); P=0.0024
BRAIN^[53]^	III期 RCT	埃克替尼 vs 放化疗	158	-	5% vs 4%	10.0 vs 4.8 HR=0.56 (0.36-0.90); P=0.014

^a^：如无特殊标注这里专指cEFR人群，cEFR指的是中枢神经系统可评估反应集。这一分析组专门包含那些在基线脑部扫描中至少有一个可测量的CNS病变的患者。与cFAS（中枢神经系统全面分析组）不同，cEFR仅包括那些具有可测量目标CNS病变的患者。评价奥希替尼与奥希替尼+化疗的疗效，使用cEFR数据集确实是更好的选择。这是因为cEFR专注于那些具有可测量CNS病变的患者，可以更准确地评估治疗对这些特定病变的反应，从而提供更具针对性的疗效数据；^b^：指脑转移亚组；^c^：包含不可测量的脑转移病变患者；^d^：采用RANO-BM标准。 EGFR-TKIs：表皮生长因子受体-酪氨酸激酶抑制剂；CNS：中枢神经系统；ORR：客观缓解率；CR：完全缓解；PFS：无进展生存期；NR：未达到；HR：危险比。

对于*EGFR*阳性脑转移的靶向治疗除考虑TKIs代际差别外，颅脑BBB的药物渗透率也应作为脑转移靶向治疗的决定因素之一。如奥希替尼可有效透过BBB，优于第一、二代TKIs^[71]^；佐利替尼由于不受BBB上P蛋白外排作用的影响，颅内浓度很高^[14]^。另外起始更高的TKIs剂量也可能提升颅内浓度，进而提升疗效。INCREASE研究^[72]^中，对基线脑转移伴L858R突变的患者，埃克替尼（加倍剂量组）中位PFS达14.3个月；但这一起始剂量增加疗法样本量有限仍需后续更多研究的证实。

除了考量颅内药物浓度，EGFR-TKIs治疗后能否达到CR也是初始靶向治疗的一个关键指标。FLAURA2研究^[73]^基线伴中枢神经系统转移的亚组中，奥希替尼联合化疗组对比奥希替尼单药组的中位PFS显著更长（24.9 *vs* 13.8个月，HR=0.47）。在进一步的颅内病灶分析中，奥希替尼联合化疗组和奥希替尼单药的CR率分别达到59%和43%^[66]^。提示接受靶向+化疗一线治疗可使相当一部分患者达到CR，降低颅脑局部治疗的必要性。

EGFR/MET双特异性抗体埃万妥单抗（Amivantamab）也被证实可进一步延缓颅内转移复发风险。MARIPOSA研究^[21]^表明，埃万妥单抗联合拉泽替尼（Lazertinib）（“2+1”强化）相比奥希替尼进一步降低*EGFR*阳性（经典突变）NSCLC脑转移患者复发或死亡风险达31%（HR=0.69, 95%CI: 0.53-0.89, *P*=0.005）。尽管埃万妥单抗疗效更优，但严重不良反应（≥3级）的风险也显著增加（75% *vs* 43%）。应当注意到，埃万妥单抗是大分子单克隆抗体，尽管有一定的颅内活性（不确定是否主要因拉泽替尼产生），对BBB的穿透能力可能有限，MARIPOSA研究目前观察到的死亡例数不足，埃万妥单抗组OS尚无法估计（not evaluated, NE），尚不能表明埃万妥单抗在*EGFR*阳性NSCLC脑转移患者中的独特优势。综合评估获益与风险、可及性后，协作组认为奥希替尼等第三代EGFR-TKIs单药仍应作为优选方案。

除了*EGFR* T790M，*MET*扩增或过表达也是*EGFR*阳性患者TKIs耐药后的常见继发突变。对于EGFR-TKIs治疗耐药的NSCLC患者，联合MET-TKIs已初步被证实可提升ORR^[74]^，但针对脑转移患者的数据有限。伯瑞替尼（Vebreltinib）I/II期研究数据^[75]^显示，联合第三代EGFR-TKIs在经TKIs治疗失败合并*MET*扩增的脑转移患者中，ORR可达66.7%。此外，INSIGHT 2研究^[76]^脑转移亚组结果也提示，继发*MET*扩增的NSCLC患者在第三代TKIs基础上联合特泊替尼（Tepotinib）后颅内ORR为29.2%，颅内DCR达79.2%，25%的脑转移患者达到颅内CR。

由于驱动基因阳性脑转移的发生与VEGF的高表达有关^[77]^，近年来多项研究评价了抗血管生成药物在NSCLC脑转移一线联合EGFR靶向治疗或后线治疗中的作用。ARTEMIS-CTONG1509研究^[78]^显示，抗血管生成药物[贝伐珠单抗（Bevacizumab）]联合EGFR-TKIs（厄洛替尼）作为NSCLC脑转移患者一线用药相比单纯TKIs组中位PFS进一步延长（17.9* vs* 11.1个月，*P*=0.008）。此外，HARMONi-A研究^[79]^也提示，对于EGFR-TKIs耐药、基线有脑转移的患者，在化疗基础上联合依沃西单抗（Ivonescimab，VEGF和PD-1的双特异性抗体）相比安慰剂提升了PFS（5.75 *vs* 4.14个月，HR=0.40）。鉴于此，对靶向治疗耐药的*EGFR*突变NSCLC脑转移患者，抗血管生成药物联合化疗也是可考虑的治疗策略之一。

**推荐8：对于EGFR经典突变的NSCLC脑转移初始靶向治疗，推荐优选以下方案：奥希替尼±化疗（1B），伏美替尼（Furmonertinib）（1B），贝福替尼（Befotertinib）（1B），阿美替尼（Almonertinib）（1B），佐利替尼（2A），埃克替尼（2A）；也可考虑选择埃万妥单抗联合拉泽替尼方案（2B）。**

**推荐9：对于已使用第一、二代EGFR-TKIs的EGFR经典突变的NSCLC脑转移患者出现T790M突变，推荐更换为颅内活性较高的第三代TKIs药物（1B）。**

**推荐10：对于已使用EGFR-TKIs的EGFR经典突变的NSCLC脑转移患者出现继发MET扩增相关耐药，可考虑联合Ib类MET-TKIs（2C）。**

**推荐11：对靶向治疗耐药的EGFR突变NSCLC脑转移患者，可考虑依沃西单抗联合化疗（2B）。**

##### 3.2.1.2 *EGFR* ex20ins和PACC突变的靶向治疗

据不完全统计，*EGFR* ex20ins突变至少有122种，具有高度异质性，因此单独使用经典的EGFR-TKIs在*EGFR* ex20ins突变患者中的敏感性不佳（ORR在0%-28%），即便使用了较高剂量^[80]^；因此，目前基于铂类化疗仍然是*EGFR* ex20ins突变NSCLC的一线治疗方法^[81]^。但随着舒沃替尼（Sunvozertinib）和埃万妥单抗新循证的出现，使得靶向治疗有望作为一线联合治疗。

WU-KONG6 II期临床试验^[82]^评价了舒沃替尼在中国*EGFR* ex20ins突变阳性的、局部晚期或转移性NSCLC患者中的疗效与安全性。受试者均经过铂类化疗，基线时31例（32%）合并脑转移，舒沃替尼治疗后的ORR为48%，提示舒沃替尼用于*EGFR* ex20ins阳性NSCLC脑转移的二线治疗有一定的颅内活性，具有一线治疗*EGFR* ex20ins突变的潜力；目前相关随机III期临床试验正在进行中。埃万妥单抗虽然在其I期关键临床研究CHRYSALIS中取得了*EGFR *ex20ins患者的高反应性结果，但可惜的是没有纳入脑转移患者。

除了*EGFR* ex20ins，对于*EGFR *PACC突变，奥希替尼等第三代TKIs药物可能依然有效，但仍需更多的研究证实。ARTICUNO研究中^[83]^，纳入的86例患者均携带非常见*EGFR*突变（G719X、L861Q、S768I点突变），其中30例合并脑转移，使用奥希替尼作为一线或二线治疗后颅内ORR依然有58%，颅内中位PFS为9个月。

在一项随机、全球、多中心、I/II期临床研究中，伏美替尼在一线治疗*EGFR *PACC突变型NSCLC患者的全球Ib期FURTHER研究数据^[84]^显示，在其240 mg *qd*剂量组中，最佳ORR高达81.8%，在伴随中枢神经系统转移的患者中，颅内ORR、DCR、CR率分别为46.2%、84.6%和38.5%，且耐受性良好。这一研究为*EGFR* PACC突变脑转移一线靶向治疗提供了新的治疗选择。

**推荐12：对EGFR ex20ins突变的NSCLC脑转移患者的二线靶向治疗，推荐舒沃替尼治疗（1B）。**

**推荐13：对EGFR PACC突变的NSCLC脑转移患者的一线靶向治疗，可考虑高剂量伏美替尼治疗（2C）。**

#### 3.2.2 *ALK*融合

克唑替尼（Crizotinib）是最早被批准用于*ALK*阳性NSCLC的靶向药物，但通常在治疗后几个月内，部分患者会出现耐药现象，而且相比化疗生存期没有显著改善^[85,86]^。后续针对耐药性和更优的靶点结合力，第二代[代表药物为阿来替尼（Alectinib）]和第三代ALK-TKIs[代表药物为洛拉替尼（Lorlatinib）]相继面世。第三代相比第二代分子量更小、亲脂性更高、受外排影响小，因此可有效地穿透BBB^[87,88]^。第二、三代药物恩沙替尼（Ensartinib）^[89]^、布格替尼（Brigatinib）^[90]^、阿来替尼^[91]^、洛拉替尼^[92]^与克唑替尼有头对头的NSCLC一线治疗用药比较，PFS和/或OS结果均优于克唑替尼，一定程度上解决了耐药问题。

针对脑转移，克唑替尼对中枢神经系统的渗透率较差^[93]^，第二、三代ALK-TKIs药物的颅内活性增加，进而改善预后。eXalt3研究^[89]^中，恩沙替尼治疗*ALK*阳性脑转移亚组中的中位PFS为11.8个月，优于克唑替尼（7.5个月，HR=0.55，*P*=0.05）。近年来我国也出现了以伊鲁阿克（Iruplinalkib）、依奉阿克（Envonalkib）为代表的新型ALK抑制剂，在脑转移人群中也表现出较高的颅内ORR，并延长了PFS，但和其他第二代ALK抑制剂一样，尚缺少长期随访数据^[94,95]^。相比之下，洛拉替尼的CROWN研究^[96]^和阿来替尼的ALEX研究^[97]^已经随访超过5年，均有超过60%的受试者（包括脑转移）实现长生存。在CROWN研究^[47]^中，洛拉替尼经过5年研究者随访，较克唑替尼组至颅内进展风险降低97%，5年无颅内进展率为83%，在基线可测量脑转移患者中，随访5年ORR为92%，颅内CR为58%，整体人群PFS率为60%，提示洛拉替尼在PFS方面有较大优势。*ALK*阳性NSCLC一线ALK-TKIs治疗脑转移疗效数据见表3^[47,89,91,95,98⇓-100]^。

**表3:** ALK+ NSCLC一线ALK-TKIs治疗脑转移疗效数据

研究名称	研究用药	脑转移亚组 样本量	基线有脑转移患者（HR）	颅内ORR （%）	颅内DOR （月）	基线有脑转移 患者中位PFS（月）
ALEX^[91]^	阿来替尼	64	0.37	59	NR（17.3-NR）	25.4
eXalt-3^[89]^	恩沙替尼	47	0.55	63.6^a^	NR	11.8
ALTA-1L^[98]^	布格替尼	47	0.25（0.14-0.46）	78^a^	27.1（16.9-42.8）	24
INSPIRE^[99,100]^	伊鲁阿克	38	0.21	90.9^a^	23.8（9.2-NE）	26.3
TQ-B3139-III-01^[95]^	依奉阿克	43	NE	78.9^a^	25.8	NE
CROWN（INV）^[47]^	洛拉替尼	35	0.08	92^a^	NR	NR

^a^：可测量的脑转移病变。 DOR：缓解持续时间；NE：无法估计。

因此，*ALK*阳性NSCLC应首选有长生存证据的洛拉替尼和阿来替尼。当洛拉替尼、阿来替尼不可及或无法耐受时，可考虑其他第二代ALK抑制剂[恩沙替尼、布格替尼、塞瑞替尼（Ceritinib）、伊鲁阿克、依奉阿克等]作为替代药物。

**推荐14：对于初治的ALK阳性NSCLC脑转移患者，优选洛拉替尼（1B）、阿来替尼（1B）靶向治疗，恩沙替尼、布格替尼、塞瑞替尼、依奉阿克、伊鲁阿克等均可考虑（2B），克唑替尼仅应在其他第二、三代ALK-TKIs不可及的情况下选用（3B）。**

当第一代ALK抑制剂出现耐药或颅内疾病进展时，应首先更换新型ALK-TKIs而不是立即起始化疗。ALUR研究^[101]^证实，阿来替尼替换克唑替尼中位PFS显著优于化疗（10.9 *vs* 1.4个月），在基线有可测量脑转移的亚组（*n*=24）中，阿来替尼的颅内ORR为66.7%[包含2例CR和14例部分缓解（partial response, PR）]，而化疗组无缓解病例。对克唑替尼（±化疗）治疗*ALK*阳性NSCLC耐药的脑转移亚组患者，也有多项研究^[102,103]^提示可以后线使用洛拉替尼，颅内ORR仍可维持在80%以上；提示克唑替尼替尼耐药的*ALK*阳性脑转移患者可考虑改换后代ALK抑制剂。至于第二、三代能否在耐药后互换，洛拉替尼有数据^[104]^表明，对替代后线治疗既往使用≥1种第二代ALK-TKIs±克唑替尼的患者，ORR为39.6%，中位PFS为6.6个月，基线可测量脑转移患者颅内ORR为56.1%。此外，有观点认为约一半ALK-TKIs耐药是“非ALK依赖性”所致，即引发EGFR、MET等其他信号通路的共激活^[105]^，但联合其他相应的靶向治疗仅有个案应用报道，获益不确定，亟待后续研究进一步明确。因此，协作组认为，在第二、三代ALK-TKIs耐药后的全身治疗可考虑联合化疗，特别是对于那些对靶向治疗无反应或已经发生广泛转移的患者。也有研究^[106]^建议联合免疫治疗，但协作组认为免疫治疗的反应率不高（8%），也缺乏理论支持。

**推荐15：ALK阳性NSCLC脑转移患者在应用克唑替尼耐药后，如果无基因检测提示继发旁路激活的情况下可改换洛拉替尼（1B）或阿来替尼（2B）。**

#### 3.2.3 *ROS1*重排

ROS1和ALK生理作用类似，近一半的氨基酸序列是一致的，ATP结合位点的同源性更是超过了3/4^[107]^，这种相似性使得部分ALK抑制剂（如克唑替尼）也兼具抑制ROS1的疗效。尽管*ROS1*阳性患者的中枢神经系统转移发生率低于ALK，但预后和靶向治疗后的疗效是相似的^[108]^。

克唑替尼是首个在NSCLC获批的ROS1-TKIs，奠定了*ROS1*重排NSCLC患者的治疗基石。相比克唑替尼，恩曲替尼（Entrectinib）、瑞普替尼（Repotrectinib）、安奈克替尼（Unecritinib）和他雷替尼（Taletrectinib）对*ROS1*的选择性更优，表现出更优的颅内ORR和PFS，并已在中国批准上市。

恩曲替尼在一线治疗*ROS1*融合阳性NSCLC脑转移患者中，中位PFS为14.9个月，中位OS长达28.3个月，且安全性良好^[109]^；STARTRK系列和ALKA研究汇总分析也证实恩曲替尼具有较高的颅内ORR^[110]^。TRIDENT-1试验^[111]^中，瑞普替尼在具有可测量中枢神经系统转移的初治患者中显示出88%的颅内ORR，对于接受过其他ROS1-TKIs治疗的患者，颅内反应率依然有42%。II期TQ-B3101研究^[112]^显示安奈克替尼整体人群ORR达81.08%；对于伴脑转移患者的ORR为72.73%，中位OS为28.22个月；在常见*ROS1*融合（CD74）及既往化疗人群中疗效较整体人群更佳。

他雷替尼是另一款国产新型ROS1-TKIs，TRUST-I研究^[113]^和TRUST-II研究^[114]^证实，在具有可测量基线脑转移患者中，不但一线治疗后的颅内ORR高（分别为87.5%和66.7%），而且在二线治疗中（即克唑替尼或恩曲替尼经治）的颅内ORR也维持在较高水平（分别为73.7%和61.7%）；提示后线治疗可考虑使用他雷替尼，但仍需更多III期临床研究进一步明确。

有真实世界研究^[115]^发现，克唑替尼和恩曲替尼在*ROS1*阳性NSCLC中的疗效相当，即便较新的ROS1抑制剂在抗肿瘤^[116]^和中枢神经系统活性^[117]^方面更优，但目前缺少上述ROS1抑制剂间头对头的临床比较研究。

**推荐16：ROS1阳性NSCLC脑转移患者初始用药可考虑恩曲替尼（1B）、瑞普替尼（1B）、他雷替尼（1B）或安奈克替尼（1B）；上述药物如不可及，克唑替尼（2B）可作为替代用药。**

目前仍缺少*ROS1*阳性NSCLC脑转移二线治疗的循证。如克唑替尼耐药，可进行基因检测，如无其他突变，可考虑尝试更换其他ROS1抑制剂（如他雷替尼），也可根据NSCLC全身进展情况考虑化疗，后续仍需积极进行临床研究以明确相关治疗路径。

#### 3.2.4 *NTRK*融合

在NSCLC脑转移有循证支持的TRK抑制剂目前有拉罗替尼（Larotrectinib）和恩曲替尼。拉罗替尼是兼具高选择性和中枢活性的TRK抑制剂。包含2项拉罗替尼研究的汇总分析^[118]^发现，在纳入的32例*NTRK*阳性的NSCLC中有12例合并脑转移，经拉罗替尼治疗后ORR为67%（8/12）。由于*NTRK*和*ROS1*也有一定的同源性，因此恩曲替尼也能用于*NTRK*阳性NSCLC患者。恩曲替尼颅内ORR在60%以上（9/14），中位颅内PFS为32.7个月^[119]^；但总的来说，NTRK靶向药物涉及的多为泛肿瘤的篮子研究，受试者纳入没有严格限制，协作组认为目前针对*NTRK*融合NSCLC脑转移靶向治疗循证虽然有限但获益明确，因此及早启用NTRK靶向治疗应是合理的。

**推荐17：对NTRK阳性初治NSCLC脑转移患者，可考虑应用恩曲替尼（1B）或拉罗替尼（1B）靶向治疗。**

#### 3.2.5 *RET*融合

*RET*基因融合往往发生于临床分期较晚的患者，因此*RET*融合虽在NSCLC的发生率不高，但32.2%的*RET*融合NSCLC患者在初诊时伴有脑转移^[120]^，这一比例在我国更高（40.3%）^[121]^。

塞普替尼（Selpercatinib）和普拉替尼（Pralsetinib）是目前临床应用最多的选择性RET抑制剂。LIBRETTO-431研究^[48]^比较了一线塞普替尼与标准治疗[含铂化疗±帕博利珠单抗（Pembrolizumab）]对*RET*融合阳性NSCLC患者的疗效和安全性；基线脑转移亚组（*n*=42）中，塞普替尼颅内ORR为82%，其中6例达到CR，12个月的颅内持续应答率为72%；并在东亚人群中显示出较高的颅内ORR（81%）^[47,122]^。普拉替尼也显示出同等水平的颅内活性，ARROW研究^[123]^中国NSCLC脑转移亚组显示，普拉替尼一线治疗ORR为85.7%，DCR为85.7%，但样本量较少（*n*=7）。

**推荐18：RET融合阳性的初治NSCLC脑转移患者，一线全身治疗推荐高选择性RET抑制剂，如塞普替尼（1B）和普拉替尼（2B）。**

对于RET-TKIs耐药的脑转移患者，建议先明确有无其他基因突变，个例报道^[124]^提示可考虑塞普替尼治疗，若塞普替尼耐药脑转移进展患者建议结合临床情况进行处理。

#### 3.2.6 *KRAS* G12C突变

与其他*EGFR*等靶向药物不同，针对*KRAS* G12C的靶向药物不是针对酪氨酸激酶的“替尼（-tinib）”而是针对GTP酶的“拉西布（-rasib）”。由于既往认为GTP酶的结构难以成药，即便*KRAS* G12C突变在NSCLC发生率不低，但直到近年其靶向治疗研究才逐渐开展，因此大多用于已经接受过放疗或手术的*KRAS* G12C突变NSCLC患者的二线治疗。最早在国外上市的两款*KRAS* G12C靶向药物——索托拉西布（Sotorasib）和阿达拉西布（Adagrasib），虽然有脑转移的证据，但目前均未在中国上市。

目前，我国已有两款新上市的*KRAS* G12C抑制剂——氟泽雷塞（Fulzerasib）和格索雷塞（Garsorasib），获批适应证均为*KRAS* G12C突变晚期NSCLC的二线治疗。氟泽雷塞在中国NSCLC人群中的关键II期临床研究中^[125]^，脑转移亚组（*n*=35，其中11例曾接受过颅脑放疗）经氟泽雷塞后线治疗后，ORR为48.6%，与整体人群无差异（高于索托拉西布和阿达拉西布既往公布的数据），PFS和OS分别为6.4和10.9个月，颅内CR为22.6%，颅内非可测量靶病灶的DCR为96.8%。格索雷塞在其I和II期临床研究中，作为二线及以上治疗*KRAS* G12C突变晚期NSCLC患者的ORR分别为17%^[126]^和61.1%^[127]^。

此外，近期还有研究^[128]^提出*EGFR*与*KRAS* G12C抑制剂联合治疗的策略，因为*KRAS* G12C抑制剂阻断RAS通路后，可能导致EGFR再激活。KROCUS研究^[129]^初步结果显示，氟泽雷塞联合西妥昔单抗治疗基线时有脑转移且*KRAS* G12C突变阳性的NSCLC患者，总体ORR达到了80%；其中，71.4%的脑转移患者实现了病理缓解。尽管样本量有限，鉴于目前*KRAS* G12C突变靶向治疗颅内ORR和PFS欠佳，这一联合疗法无疑带来了新选择。

**推荐19：对KRAS G12C突变的NSCLC脑转移患者，推荐氟泽雷塞（1B）、格索雷塞（1B）二线治疗，鼓励患者进行一线联合的临床研究试验。**

#### 3.2.7 *MET*ex14跳跃突变

尽管临床上可用的针对*MET*突变的药物数量不断涌现，但脑转移人群的证据不多，集中在共识或指南优选的Ib类MET抑制剂循证。大多数Ib类抑制剂均能够有效通过BBB^[130]^。一项纳入68例*MET*ex14跳跃突变脑转移患者使用卡马替尼（Capmatinib）的真实世界研究^[131]^显示，55例患者接受一线卡马替尼治疗颅内缓解率为87.3%；其中20例未经放疗的患者颅内缓解率为85%，两者中位PFS均为14.1个月。KUNPENG研究^[132]^证实，对晚期*MET*ex14跳跃突变的中国NSCLC患者，伯瑞替尼治疗晚期*MET*ex14跳跃突变的中国NSCLC患者的中位PFS为14.1个月，OS为20.7个月，其中脑转移亚组（*n*=5）中位PFS较短，为6.4个月，OS为17.9个月。值得一提的是，伯瑞替尼是目前唯一具有肺癌及脑胶质瘤适应证的MET抑制剂。GEOMETRY mono-1研究^[133]^中，在接受卡马替尼治疗的13例脑转移患者中，有7例观察到颅内反应（其中3例有放疗史）；中国Geometry-C研究^[134]^纳入4例基线脑转移的患者，2例患者颅内病灶达到CR，2例患者颅内病灶为PR。VISION研究（单臂II期）脑转移亚组结果^[135]^显示，通过RANO-BM标准进行评估，特泊替尼在初治和经治*MET*ex14跳跃突变患者中的颅内ORR为66.7%，颅内DCR为88.4%，颅内中位PFS为20.9个月，显示出持久的抗肿瘤活性。此外，赛沃替尼（Savolitinib）^[136]^和谷美替尼（Glumetinib）^[137]^也表现出一定颅内活性。一项IIIb期赛沃替尼研究^[138]^中，纳入了79例经治的*MET*ex14跳跃突变的患者（二线治疗组）和87例初治患者（一线治疗组），结果显示赛沃替尼一线治疗组和二线治疗组的ORR分别为62.1%和39.2%，中位PFS分别为13.7和11.0个月，中位OS尚未成熟。

应该注意到，由于突变发生率本身较低，这些Ib类MET抑制剂研究的脑转移亚组例数偏低，治疗*MET*ex14跳跃突变NSCLC脑转移的证据虽然较多，但统计效能往往不足，也没有对照组。因此，协作组认为*MET*ex14跳跃突变脑转移的一线靶向治疗，可参考*MET*ex14跳跃突变NSCLC整体人群的推荐，优先选择有颅内活性证据的药物，如谷美替尼、伯瑞替尼、特泊替尼、卡马替尼和赛沃替尼（有一线用药的证据，但是没有获批适应证）。如Ib类药物不可及，也可选择克唑替尼、恩沙替尼等Ia类药物替代。对于初始MET抑制剂疗效不佳或耐药的患者，考虑到*MET*ex14跳跃突变的耐药机制目前还未完全明确，及早进行二次基因检测，明确是否有新发突变，如没有新发突变，建议考虑化疗而不是换用其他MET抑制剂可能是合理的。

**推荐20：对METex14跳跃突变的NSCLC脑转移患者一线或二线靶向治疗，优先选择有颅内活性证据的Ib类MET抑制剂，如伯瑞替尼、特泊替尼、卡马替尼、谷美替尼和赛沃替尼（2B）。**

#### 3.2.8 *HER2*突变

NSCLC中，*HER2*基因异常主要表现为基因扩增/过表达和突变（主要为ex20ins突变）的形式，而后者更为常见^[139]^。*HER2*阳性NSCLC脑转移靶向治疗的重点是针对*HER2*突变（特别是针对20ins或YVMA插入）的抗体偶联药物（antibody-drug conjugate, ADC）或TKIs。

HER2-ADC方面，已经上市的有恩美曲妥珠单抗（Ado-trastuzumab emtansine, T-DM1）和德曲妥珠单抗（Trastuzumab deruxtecan, T-DXd）。恩美曲妥珠单抗虽然在NSCLC HER2靶向治疗的临床研究开展较早，但很少涉及脑转移人群^[140]^。德曲妥珠单抗则后来者居上，在DESTINY-Lung02研究的脑转移亚组（*n*=20）中，显示出良好的疗效，ORR为64.3%（9/14），其中包括7例CR，颅内反应持续时间为55.7个月。此外，DESTINY-Lung01研究^[141]^中也纳入了33例伴有脑转移的患者，其ORR为54.5%，中位PFS为7.1个月，中位OS为13.8个月。近期，中国国家药品监督管理局批准了德曲妥珠单抗用于*HER2*突变晚期不可切除或转移性NSCLC二线及以上治疗的适应证。

吡咯替尼（Pyrotinib）是我国应用广泛的一种HER2-TKIs。在一项II期多中心研究^[142]^中，纳入化疗经治后的*HER2*阳性NSCLC患者，二线应用吡咯替尼后整体ORR为30%，脑转移亚组略低（25%），整体的PFS和OS分别为6.9和14.4个月。也有研究^[143]^指出将吡咯替尼与阿帕替尼（Apatinib，小分子VEGFR-2抑制剂）联合使用，提升了基线伴脑转移*HER2*+患者（*n*=13）的ORR至53.8%。TKIs新药方面，吡咯替尼之后出现了在HER2靶点高选择性上更优的TKIs，疗效和安全性更高，循证样本量也更多。首先取得我国“突破性治疗品种名单”的是BAY 2927088，它是一种可逆性TKIs，在临床前模型中可有效抑制*HER2*激活突变。SOHO-01的扩展队列（I/II期）证实，对未经靶向治疗的二线*HER2*阳性NSCLC患者，BAY 2927088整体ORR提升至72.1%，特别针对YVMA插入患者ORR高达90.0%（27/30），脑转移亚组ORR为62.5%（5/8）^[144]^，针对该人群晚期一线治疗III期研究在全球范围内进行。这一新型HER2-TKIs将很快在我国上市。Zongertinib也是高选择性的口服HER2-TKIs，并保留了野生型EGFR的活性。Beamion LUNG-1研究^[145]^评估了其在二线治疗*HER2*阳性NSCLC的疗效与安全性，整体人群ORR为72%，DCR为95%。在脑转移亚组（*n*=54）中，整体ORR达70%，DCR高达94%；与整体人群保持一致。大多数不良事件为轻度可控，因不良事件导致停药的比例仅为3%^[146]^，提示Zongertinib在*HER2*突变NSCLC及脑转移患者中具有良好的抗肿瘤活性和安全性，后续Beamion LUNG-2 III期试验将验证在一线治疗中的潜力。Zongertinib也累积了较多中国患者数据，最近获得我国药监局“突破性疗法”认定，用于*HER2*突变、晚期不可切除或转移性NSCLC患者的二线或后线治疗。

综上，无论是HER2-TKIs还是ADC，目前大多集中在二线或后线用药，但协作组认为，可以开展临床试验，探究在其他治疗基础上及早联合HER2-TKIs或ADC的必要性和长期预后。

**推荐21：鼓励HER2突变阳性NSCLC脑转移患者参加一线HER2-TKIs临床研究，二线及后线靶向治疗推荐德曲妥珠单抗（1B）或吡咯替尼（2C）。**

#### 3.2.9 *BRAF* V600突变

由于单一使用BRAF抑制剂达拉非尼（Dabrafenib）很容易出现耐药，通常联用MEK抑制剂曲美替尼（Trametinib），目前这一组合已成为NSCLC *BRAF* V600突变的标准靶向治疗。虽然该组合疗法已被证明可以改善转移性*BRAF* V600突变NSCLC患者预后，但在脑转移人群中的证据不足。达拉非尼可穿透BBB，脑脊液中浓度高于同类药物[维莫非尼（Vemurafenib）]^[147]^。在一项达拉非尼和曲美替尼治疗*BRAF* V600E突变NSCLC研究^[148]^的脑转移亚组中（*n*=34），联合治疗后观察到25例（80.6%）达到客观缓解，中位PFS为7.5个月，中位OS达24.1个月。在达拉非尼和曲美替尼II期关键性临床研究^[149]^中，虽排除活动性脑转移，但仍纳入了3例基线脑转移病灶为非靶病灶的患者，无论一线还是后线最佳疗效均达到PR，治疗后未有新发脑转移进展。此外，还有个例报道^[150,151]^探索了达拉非尼、曲美替尼用于一线治疗*BRAF* V600突变脑转移患者脑部病灶均达到完全缓解。

康奈非尼（Encorafenib）联合比美替尼（Binimetinib）也表现出一定的疗效，PHAROS研究^[152]^是一项II期开放标签临床试验，评估了康奈非尼联合比美替尼在*BRAF* V600E突变转移性NSCLC患者中的疗效。该研究共纳入98例患者（59例初治，39例经治），其中脑转移发生率分别为7%和10%。结果显示，初治组的ORR为75%（95%CI: 62%-85%），经治组为46%（95%CI: 30%-63%）；中位持续缓解时间（duration of response, DOR）分别为NE和16.7个月（95%CI: 7.4-NE）；中位PFS分别为NE和9.3个月（95%CI: 6.2-NE）。另有研究^[153]^提示，联合康奈非尼加比美替尼的*BRAF* V600突变的NSCLC患者中8例合并脑转移，其中有4例显示出抗肿瘤活性（所有4例为未接受过治疗的患者，或者为既往接受过治疗且均无客观反应），表现为CR或PR。

**推荐22：对BRAF V600突变的NSCLC脑转移患者，可考虑达拉非尼联合曲美替尼治疗（2C）。**

### 3.3 外科手术

目前，由于靶向治疗的发展，无需急症干预的驱动基因阳性NSCLC脑转移灶的外科手术已被推迟，特别是比传统放疗更为精准的SRS的出现，也取代了部分外科手术。但驱动基因阳性患者（特别是对靶向药物敏感的）相比驱动基因阴性患者预期寿命较长，给了外科手术更长的干预时间窗，而且靶向治疗后“缩瘤”，也为体积较大的脑转移瘤的完整切除、达到局部治愈提供了可能性。另外，当脑转移灶出现放射性坏死时，手术切除坏死灶也是可以考虑的。可见，外科手术仍是NSCLC脑转移综合治疗的重要组成部分，除了可以迅速缓解颅内占位症状和脑疝急症风险，还可显著延长患者的生存。一项776例NSCLC伴脑转移患者回顾性队列研究^[154]^发现，外科手术加放疗的患者中位生存期为26.25个月，显著优于仅接受放疗的患者（14.49个月，*P*<0.001）。对靶向治疗和放疗后颅内病灶没有达到CR的脑转移患者是否应积极行外科干预，目前没有明确的定论，但是对于靶向药物治疗后脑转移灶进展且有症状的患者，推荐开颅手术或者立体定向活检术，对脑转移灶进行分子病理学检测，指导下一步治疗。此外，也有报道^[155]^提示，如果只有脑转移，颅内切除转移瘤后再行肺部原发肿瘤切除等综合治疗，可进一步改善生存。

因此，协作组推荐手术前进行多学科讨论，充分评估患者是否能从手术中获益。此外，外科手术适应证与其他脑转移瘤并无显著差异，手术能否进行仍主要取决于颅内转移病灶的数量、位置、大小、水肿以及患者的整体状况等，特别是有明显症状或较大肿瘤负担的患者，但具体指征尚未统一；手术方式的推荐也与NCCN、ASCO-SNO-ASTRO、EANO-ESMO等指南的相关推荐并无差异，本指南不再赘述。

**推荐23：对于靶向药物治疗后脑转移灶进展且有症状的患者，可考虑开颅手术或者立体定向活检术，对脑转移灶进行分子病理学检测，指导下一步治疗（2D）。**

### 3.4 放疗

相比WBRT时代，近年来针对脑转移瘤的放疗技术显著改进，立体定向放射治疗（stereotactic radiation therapy, SRT）开始在临床普及，有效降低了放疗副作用。但对于驱动基因阳性的NSCLC脑转移患者，目前放疗仍缺乏前瞻性临床研究证据，也未有证据表明驱动基因阳性与否是疗效的独立影响因素，因此本章节推荐是基于包含NSCLC在内的脑转移瘤的循证做出的。

#### 3.4.1 NSCLC脑转移放疗适应证

如前所述，对于无症状的NSCLC脑转移患者，局部放疗可以延后，使用TKIs后出现脑部寡进展或有症状脑转移时推荐使用局部放疗^[156]^。此外，有研究^[157]^提示，在外科切除脑转移瘤后进行放疗可以有效抑制肿瘤复发且可耐受，故推荐术后使用放疗延缓复发。

#### 3.4.2 放疗方式和剂量的选择

脑转移瘤的放疗方式主要有两种：WBRT和更为精准的SRT。SRT又分为单次的SRS和多分次的SRT，前者通常为单次大剂量照射，剂量范围一般为16-30 Gy，后者通常在3-5次之间进行。

放疗方式的选择主要取决于转移瘤的数量、大小和分布。

在转移瘤的数量上，多项随机对照研究^[158⇓-160]^评估了WBRT联合SRS与单独使用SRS在脑转移瘤（包括NSCLC）患者中的疗效，这些研究主要纳入了1-3个脑转移瘤的患者，结果发现，尽管将WBRT联合SRS可以改善颅内控制，但并未改善生存率。相反，WBRT的应用与神经认知功能的下降和生活质量的恶化相关^[161]^。基于此，协作组认为对于表现状态良好且寡转移的（有1-3个转移灶）、包膜完整的NSCLC脑转移瘤患者，建议采用SRS或SRT治疗方案。超过3个转移灶的患者放疗方案，目前仍有争议^[162]^，协作组专家推荐使用WBRT，剂量为30 Gy/10 F或37.5 Gy/15 F^[163,164]^；但若转移灶距海马>5 mm且预期寿命>4个月，应考虑行海马保护，并同时应用美金刚治疗^[165]^；如症状无法缓解可考虑联合SRS/SRT。对于4-10个脑转移灶的患者放疗方案，有条件的医疗中心也可以使用SRS/SRT^[162]^。

在转移瘤的大小上，对于直径≤4 cm且远离重要脑组织结构，推荐行SRS治疗，但肿瘤最大直径超过3 cm时应用SRS应慎重。推荐依据病灶最大直径制定SRS放疗剂量^[166]^，具体如表4所示。对于产生占位效应和/或大小超过4 cm的转移瘤，则不适宜首选放疗，建议进行MDT讨论，行手术切除。如果手术不可行，可使用SRT（27 Gy/3 F或30 Gy/5 F）。SRS通常不推荐，因为较大的肿瘤更容易导致放射性坏死和局部控制失败^[57]^。

**表4:** 根据脑转移瘤病灶最大直径制定SRS放疗的剂量推荐

转移瘤最大直径	放疗剂量推荐
≤2.0 cm	24 Gy
2.1-3.0 cm	18 Gy
3.1-4.0 cm	15 Gy

SRS：立体定向放射外科。

在转移瘤的分布上，如果转移瘤毗邻重要脑组织结构（如脑干、视交叉），推荐行SRT^[167⇓⇓-170]^，剂量可考虑21-27 Gy分3次或25-35 Gy分5次进行。

此外，对于脑转移行外科手术者，推荐使用瘤腔补充SRS/SRT。推荐根据瘤腔体积制定SRS放疗剂量^[161]^，具体见表5。

**表5:** 根据瘤腔体积制定SRS放疗的剂量推荐

瘤腔体积	放疗剂量推荐
4.2 cm^3^	20 Gy
4.3-7.9 cm^3^	18 Gy
8.0-14.3 cm^3^	17 Gy
14.4-19.9 cm^3^	15 Gy
20.0-29.9 cm^3^	14 Gy
≥30.0 cm^3^且最大直径≤5.0 cm	12 Gy

**推荐24：对于无症状的NSCLC脑转移患者，局部放疗可以延后；对使用TKIs后出现脑部寡进展或有症状的脑转移患者，推荐尽早使用局部放疗（1A）；放疗方式的选择主要取决于转移瘤的数量、大小和分布（1A）。**

#### 3.4.3 放疗期间能否继续使用TKIs

在放疗期间，临床实践通常倾向于继续TKIs治疗。值得注意的是，脑放疗与TKIs联合使用的安全性数据尚不充分。一项前瞻性II期临床研究^[171]^显示，WBRT同步厄洛替尼的耐受性良好，没有观察到增加的神经毒性，且无患者经历4级或以上的毒性反应。基于专家共识和临床经验，目前建议在WBRT或SRS/SRT治疗期间继续TKIs治疗，但WBRT+SRS/SRT联合TKIs时需慎重。这一领域仍需更多前瞻性研究来指导临床决策，特别是在优化TKIs与放疗联合方面。

### 3.5 对症治疗

驱动基因阳性NSCLC脑转移与其他脑转移瘤的症状治疗原则一致，包括减轻脑水肿、降颅压、癫痫防治和头痛处理等。甘露醇降颅压、抗癫痫和止痛处理并无特殊，可参考神经内科相关指南进行用药，本指南重点强调NSCLC脑转移水肿的处理。

控制脑水肿是肺癌脑转移对症治疗的重点，主要是系统应用糖皮质激素，还可配合抗血管生成药物及渗透性利尿药（甘露醇±利尿剂）。糖皮质激素（主要为地塞米松）可降低血管通透性，增强BBB的完整性，有助于减轻脑水肿^[172]^。在无症状的患者中是否常规应用激素目前仍缺乏直接证据，由于放疗会加重脑水肿，放疗前后使用地塞米松可能会有所改善^[173]^。若患者有中至重度脑水肿症状，如剧烈头痛、恶心呕吐或明显局灶性神经功能障碍等，可先给予负荷剂量的地塞米松10 mg，之后以8-16 mg/d起始，可分1-2次给药。若患者症状较轻，通常不给予负荷剂量，地塞米松起始剂量可2-4 mg/d^[174]^。由于VEGF在瘤周水肿的发病机制中发挥着重要作用，抗血管生成药物贝伐珠单抗有助于减轻脑水肿^[175]^。对于需要长期使用糖皮质激素的患者，贝伐珠单抗具有糖皮质激素助减作用^[176]^。

此外，应当注意的是，NSCLC脑转移患者中常出现静脉血栓形成（venous thromboembolism, VTE）^[177]^，且驱动基因阳性患者的发生率普遍高于野生型^[178]^。因此，对血栓高危的驱动基因阳性NSCLC脑转移人群应加强VTE筛查，必要时可考虑进行抗凝治疗。

**推荐25：对出现脑水肿或颅内高压的NSCLC脑转移患者，应及时予以糖皮质激素、甘露醇治疗，必要时配合抗血管生成药物及渗透性利尿药，以减轻颅内症状（1C）。**

## 4 免责声明

协作组撰写的此份指南旨在协助相关领域的肿瘤专科医师制定临床治疗参考，仅代表协作组的专家共识和经验。本指南中的推荐仅适用于驱动基因阳性NSCLC的脑实质转移，不适用于所有肺癌和其他脑转移瘤。本指南中的推荐不应被视为完整或绝对正确，也不应被视为一成不变的标准治疗和临床实践准则，更不能替代临床专科医生的独立判断。随着循证医学的快速发展，新证据可能会在本指南撰写、修改、杂志审校排版发表期间出现，本指南中所引用的研究（2024年10月前收录于PubMed的相关文献）可能无法反映最新循证。

**指南编写专家组成员**

**组长**

支修益 首都医科大学宣武医院

王洁 中国医学科学院肿瘤医院

**执笔**

赵军 北京大学肿瘤医院暨北京市肿瘤防治研究所

胸部肿瘤内一科，恶性肿瘤发病机制

及转化研究教育部重点实验室

李晓燕 首都医科大学附属北京天坛医院

**成员**（按姓氏拼音排序）

曹宝山 北京大学第三医院

蔡修宇 中山大学肿瘤防治中心

陈麦林 北京大学肿瘤医院

陈绪珠 首都医科大学附属北京天坛医院

褚倩 华中科技大学同济医学院附属同济医院

戴朝霞 大连医科大学附属第二医院

段建春 中国医学科学院肿瘤医院

冯晨璐 首都医科大学附属北京天坛医院

胡牧 首都医科大学附属北京友谊医院

胡毅 中国人民解放军总医院

胡瑛 首都医科大学附属北京胸科医院

黄鼎智 天津医科大学肿瘤医院

李囡 北京大学肿瘤医院

李子明 上海市胸科医院

林根 首都医科大学附属北京胸科医院

刘玉良 北京大学肿瘤医院

农靖颖 首都医科大学宣武医院

商琰红 河北大学附属医院

石安辉 北京大学肿瘤医院暨北京市肿瘤防治研究所

宋天彬 首都医科大学宣武医院

苏春霞 上海市肺科医院

孙时斌 首都医科大学附属北京天坛医院

汤传昊 北京大学国际医院

汪进良 中国人民解放军总医院

王志杰 中国医学科学院肿瘤医院

吴芳 中南大学湘雅二医院

邬麟 湖南省肿瘤医院

徐蔚然 首都医科大学附属北京天坛医院

姚煜 西安交通大学第一附属医院

杨波 中国人民解放军总医院

杨萌 中日友好医院

杨雪 北京大学肿瘤医院

杨宇 哈尔滨医科大学附属第二医院

燕翔 北京大学人民医院

易福梅 北京大学第三医院

张明山 首都医科大学三博脑科医院

张燕 河北医科大学附属人民医院

赵晖 首都医科大学附属北京天坛医院

赵博 北京大学肿瘤医院

周进 四川省肿瘤医院

周清 广东省人民医院肿瘤医院

庄洪卿 北京大学第三医院

卓明磊 北京大学肿瘤医院

朱翔 北京大学医学部/北京大学第三医院

**特邀顾问**

杨学军 清华大学附属北京清华长庚医院

焦顺昌 中国人民解放军总医院肿瘤医学部

派驻第一医学中心

李琳 北京医院
